# Microbial Load of Fresh Blueberries Harvested by Different Methods

**DOI:** 10.3390/foods12051047

**Published:** 2023-03-01

**Authors:** Peien Wang, Minji Hur, Yixin Cai, Fumiomi Takeda, Lisa DeVetter, Jinru Chen

**Affiliations:** 1 Department of Food Science and Technology, The University of Georgia, Griffin, GA 30223, USA; 2 Department of Horticulture and Northwestern Washington Research and Extension Center, Washington State University, Mount Vernon, WA 98273, USA; 3 Appalachian Fruit Research Station, Agricultural Research Service, US Department of Agriculture, Kearneysville, WV 25430, USA

**Keywords:** blueberry, blueberry machine harvester, food safety, handpicking

## Abstract

Currently, more and more growers are transitioning to the use of over-the-row machine harvesters for harvesting fresh market blueberries. This study assessed the microbial load of fresh blueberries harvested by different methods. Samples (*n* = 336) of ‘Draper’ and ‘Liberty’ northern highbush blueberries, which were harvested using a conventional over-the-row machine harvester, a modified machine harvester prototype, ungloved but sanitized hands, and hands wearing sterile gloves were collected from a blueberry farm near Lynden, WA, in the Pacific Northwest at 9 am, 12 noon, and 3 pm on four different harvest days during the 2019 harvest season. Eight replicates of each sample were collected at each sampling point and evaluated for the populations of total aerobes (TA), total yeasts and molds (YM), and total coliforms (TC), as well as for the incidence of fecal coliforms and enterococci. The harvest method was a significant factor (*p* < 0.05) influencing the TA and TC counts, the harvest time was a significant factor influencing the YM counts, while the blueberry cultivar was an insignificant (*p* > 0.05) factor for all three indicator microorganisms. These results suggest that effective harvester cleaning methods should be developed to prevent fresh blueberry contamination by microorganisms. This research will likely benefit blueberry and other fresh fruit producers.

## 1. Introduction

Blueberry (*Vaccinium* spp.) consumption in the United States has steadily increased over the past few decades, primarily due to increased consumer awareness of the health benefits associated with blueberry consumption. A large quantity of blueberries is produced on a global scale to meet this increasing consumer demand [1,2]. The United States is currently the largest blueberry-producing country, with a production share of approximately 56% between 2009 and 2011 [3]. Within the United States, Washington has become the largest blueberry-producing area in the world, with *ca*. 75,000 kg of utilized production in 2020, and about half of its utilized production goes to the fresh market [4].

Blueberries for the fresh market are primarily harvested by hand during the early harvest season [2]. Handpicking favors high-quality fruit with a firmer texture and longer postharvest shelf life [5]. However, a lack of worker availability and increasing labor costs are major constraints for berry growers that rely on hand labor. Thus, growers are increasingly transitioning to the use of over-the row (OTR) machine harvesters to harvest blueberries for the fresh market [6].

During the harvesting process of a top-loading machine, blueberries are separated from bushes by the vibration of plastic beater bars mounted on rotary shaking drums. The separated fruit fall onto catching plates and adjacent hard surfaces at the bottom of the harvester [7]. The fruit roll onto horizontal conveyors that transport the fruit to the rear of the harvester, where they fall into small buckets that elevate the fruit to the top platform of the harvester. Once at the top platform, debris and leaves, clustered berries, as well as small, immature berries are separated as the blueberries fall through a fast-moving upward stream of air. The air-sorted blueberries are dropped onto a horizontal manual grading/inspection belt to remove some crushed and diseased fruit, before dropping into the harvest lugs.

Although there are several advantages associated with OTR machine harvesting, machine-harvested blueberries have internal bruise damage [8]. The hard surfaces of machine harvesters, such as the fruit-shaking beater rods and plastic-catching plates, create significant damage to the harvested berries, including increased bruising and reduced firmness compared to hand-harvested fruit [7,9]. As a result, berries harvested by mechanical harvesters have a shorter shelf life than hand-harvested blueberries [10].

To reduce the blueberry quality losses caused by mechanical harvesting, several attempts have been made to modify standard OTR harvesters by replacing the hard surfaces with softer materials [8,11,12]. An OTR machine prototype with modified fruit catching plates and intermittent soft surfaces suspended above the hard surfaces was used to harvest blueberries [8,10,11,13]. Brown et al. [8] and Sargent et al. [9] reported that the modification of the hard catch plates on a blueberry machine harvester with a soft cushioning material significantly reduced the physical impacts to the harvested berries. However, it is currently unknown whether such modifications would adversely affect the microbial safety of harvested fruit. The objective of this study was to compare the microbial load of fresh blueberries harvested using a standard OTR machine harvester, a prototype modified machine harvester with softer catching surfaces, ungloved but cleaned and sanitized hands, and hands wearing sterile gloves.

## 2. Materials and Methods

### 2.1. Sample Collection

Samples of ‘Liberty’ and ‘Draper’ northern highbush (*Vaccinium corymbosum*) blueberries were collected at two different geographic locations within a commercial blueberry farm located near Lynden, WA, in the Pacific Northwest region of the United States (latitude 48°58″38.35, longitude−122°21″27.07 [whatismygps.com, accessed on 10 January 2020], altitude 40 m [how-far.net]) in July and September of 2019. The blueberry samples were collected at three different times (9 am, 12 noon, and 3 pm) on four different harvesting days separated by two months. The average temperatures at 9 am, 12 noon, and 3 pm on the four harvest days were 18.33 °C ± 0.48, 21.67 °C ± 2.59, and 23.89 °C ± 3.60, respectively, and the relative humidity values were 89.65% ± 6.10, 72.78% ± 17.30, and 62.40% ± 22.59, respectively.

At each sampling point, eight replicates of the blueberry samples were collected using each of the four harvesting methods: ungloved but cleaned and sanitized hands, standard OTR machine harvester, and modified OTR machine harvester prototype, while the samples collected by hands wearing sterile nitrile gloves (Fisher Scientific, Pittsburg, PA, USA) served as controls. The workers on both machine harvesters also wore sterile nitrile gloves. Both types of machine harvesters were manufactured by the Oxbo International Corporation (Lynden, WA, USA). The catch surfaces and catch plates of the conventional OTR harvester were made of proprietary hard polycarbonate plastic. As for the modified OTR prototype, both hard catch surfaces and the center of each catch plate were replaced with a type of soft, food-grade elastomeric polymer within a slender polycarbonate frame left on each plate [14]. The handpicked blueberry samples were randomly collected from different locations on multiple blueberry bushes, while the berries harvested using the conventional and modified OTR machines were randomly collected from stackable berry lugs, with one sample being collected per lug. The collected blueberries were placed in sterile Whirl-Pak bags (Nasco, Fort Atkinson, WI, USA) and kept in a cooler (Rubbermaid; Newell Brands Inc, Atlanta, GA, USA) with ice packs (VWR International LLC, Radnor, PA, USA) in the field and during transportation to a laboratory in Lynden, WA, USA, for the sample processing.

### 2.2. Sample Processing and Transportation

The collected blueberry samples were stored in the cooler no longer than 1 h after each harvest time point, and they were processed immediately upon arrival at the laboratory. Each blueberry sample (25 g) taken from fruit harvested in the field was placed in a fresh, sterile Whirl-Pak bag and homogenized (Stomacher 80, Seward Ltd., West Sussex, UK) in 50 mL of sterile 0.2 M phosphate-buffered saline (PBS, pH 7.4) for 1 min at normal speed. The resulting homogenate, in a volume of 7.5 mL, was transferred to a 15 mL conical centrifuge tube (Fisher Scientific) containing 2.5 mL of 60% glycerol to make the final glycerol concentration of 15% (*v*/*v*). Each sample was vigorously mixed and then stored at −20 °C before being transported overnight by aircraft to Atlanta, GA, followed by ground transportation to our laboratory in Griffin, GA, in an insulated polystyrene foam container (Polar Tech 266C Thermo Chill Insulated Carton with Foam Shipper, 19″ × 12″ × 16″, Genoa, IL, USA) with 5 lb. of dry ice (Ice Company LLC, Lynden, WA, USA). The samples were analyzed immediately upon arrival at the laboratory on the University of Georgia Griffin Campus.

### 2.3. Microbiological Analyses

The frozen blueberry homogenates described above were thawed upon arrival at the laboratory. The individual blueberry homogenates (100 µL) were inoculated in duplicate on four different microbiological media, including tryptic soy agar (TSA), potato dextrose agar (PDA) acidified with 10% tartaric acid to pH 3.5, MacConkey agar (MAC), and enterococcus agar (EA). All the microbial media used in the study were purchased from Becton Dickinson Inc. (Franklin Lake, NJ, USA). Serial dilutions were made in sterile 0.1 M PBS when necessary, before inoculation onto the various microbiological media. The incubation condition for the TA, TC, and enterococci was at 37 °C for 24 to 48 h, fecal coliforms at 44.5 °C for 24 h, and YM at 25 °C for 48 to 72 h. The colonies were enumerated after the incubation, and the results were presented as the log colony-forming units per gram of fresh blueberry sample (log CFU/g). The detection limit of the plate count assay was 1.12 log CFU/g.

### 2.4. Statistical Analysis

A split-plot design analysis of variance (ANOVA) was used in the current study. Each sampling location with one blueberry cultivar planted was considered to be the main plot experimental unit, while the cultivar itself was the main plot factor. The sampling time and harvesting method were two factors nested within each level of the main plot, forming subplot factors. The random error term was reflected by the effect of the repeated visits, as well as by the interaction effect between the visits and different cultivars. All the microbiological data were fitted into a general linear model, and Fisher’s least significant difference test was used to separate the means (SAS, version 9.4, Statistical Analysis System Institute, Inc., Cary, NC, USA). Differences were considered significant when the *p* values were smaller than 0.05. The numbers of positive samples for the fecal coliforms and enterococci were recorded to calculate the respective incidence of these two indicators in the total number of samples collected within the same factor.

## 3. Results

According to the results of the type III test, the harvesting method was a significant (*p* < 0.05) factor influencing the counts of TA and TC, the sampling time was a significant factor influencing only the counts of YM on the sampled blueberries, while the blueberry cultivar was an insignificant (*p* > 0.05) factor for the counts of all three indicator microorganisms (Table 1). No significant interactions between or among the independent variables were observed.

The samples of the two blueberry cultivars had similar (*p* > 0.05) mean TA, YM, and TC counts (Table 2). In general, the average TA and TC counts on the sampled blueberries were low, with the mean TA count being ca. 2 log CFU/g and the mean TC count being less than 0.5 log CFU/g. In comparison, the mean YM counts were relatively higher, being in the 3 log CFU/g range in the berries from both cultivars.

On average, the berries collected at the three different time intervals had similar mean TA counts (Table 2). Similar mean YM counts were found for the 9 am and 12 noon samples, and the counts for the samples collected at these two intervals were significantly higher (*p* < 0.05) than the same count for the 3 pm samples. The 3 pm samples also had significantly lower mean TC counts compared to the 9 am samples.

The blueberries harvested using the standard OTR machine harvester and modified OTR machine harvester prototype had significantly higher (*p* < 0.05) mean TA counts than those harvested using hands with sterile gloves and ungloved but cleaned and sanitized hands (Table 2). The berry samples collected using the modified OTR prototype also had significantly higher TC counts than those harvested using the other three different methods. However, the levels of the mean YM counts for all the collected berry samples were not different (*p* > 0.05).

When the microbial counts for the berries harvested using different methods were separated by the time of the sample collection, the samples harvested using the two types of machines had significantly higher (*p* < 0.05) TA counts than those harvested using the other two methods in the 9 am and 3 pm samples (Table 3). For the noon samples, however, the samples collected using bare hands had similar TA counts to the samples collected using the other three methods. Furthermore, the samples collected using the conventional machine harvester and both bare and gloved hands had similar (*p* > 0.05) mean TC counts at the 9 am sampling points, and these counts were significantly lower than the mean TC count recovered from the samples harvested using the modified machine harvester prototype at the same time interval. For the noon samples, however, the mean TC count for the fruit harvested using the modified OTR machine harvester prototype was only significantly higher than for the two groups of handpicked fruits.

The results in Table 4 demonstrate that the samples of both blueberry cultivars collected using the two machine harvesters had significantly higher (*p* < 0.05) mean TA counts than those harvested using bare and gloved hands, an observation similar to the results of the overall statistical analysis in Table 2. The ‘Liberty’ berries harvested using the conventional machine harvester and using bare and gloved hands had similar (*p* > 0.05) mean TC counts, which were significantly lower than the TC count for the samples harvested using the modified machine harvester prototype.

No enterococci were detected in any of the collected samples, whereas seven fecal coliforms were recovered from the berry samples harvested using the modified OTR machine harvester prototype. The percentage of fecal coliform positive samples was 2.1%. Three out of the seven isolates were from the 9 am samples, and the other four isolates were from the noon samples.

## 4. Discussion

### 4.1. The Effect of Cultivars

The ‘Draper’ blueberry is a mid-season cultivar known for its firm texture, whereas the ‘Liberty’ is a mid- to late-season cultivar with outstanding flavor and good shelf life [15,16]. Different cultivars of fresh produce may have different intrinsic factors, such as the pH, water content, surface morphology, etc., which may affect the level and diversity of the microorganisms associated with fresh produce [17]. However, the results of the current study revealed no difference (*p* > 0.05) in the microbial counts of the samples of the two cultivars (Table 2). One possible interpretation of this observation is that the overall phytochemical profiles of these two cultivars might be quite similar and had an indistinguishable influence on the microbial counts for the collected blueberry samples.

Fresh produce grown in the field are susceptible to contamination by microorganisms from irrigation water, soil, manure fertilizers, insects, domestic and wild animals, and produce handlers [18,19]. Extrinsic factors such as climate conditions, geographic locations, and agricultural practices (i.e., irrigation, spraying, fertilization, etc.) may influence the introduction of foodborne pathogens into fresh produce [17]. For example, when irrigation water was inoculated with *Salmonella*, the furrow-irrigated cantaloupes (*Cucumis melo* subsp. *melo*) had a microbial load that was two to four times higher than the drip-irrigated ones [20]. Given the fact that the two blueberry cultivars sampled in the current study were grown in adjacent areas on one farm, under similar climate conditions and management they could be loaded with similar levels of microorganisms sourced from the environment.

### 4.2. YM Counts in Sampled Blueberries

Fresh fruit, such as blueberries, are rich in sugar and nutrients, have a high moisture content, and are ideal media for microorganisms to thrive on [21]. The pH of the ‘Draper’ and ‘Liberty’ blueberries was estimated to be 3.4 and 3.5, respectively [22]. A relatively low pH is favorable for the growth of acid-tolerant spoilage microorganisms such as yeasts, molds, and other fungi, which may cause blueberry rot and deterioration [23,24]. The mean count of YM in the samples collected in the current study was ca. 3.80 log CFU/g (Table 2). Slightly higher levels of YM counts were reported in several other studies in the United States. For example, a study performed in Georgia recovered 4.49 log CFU/g of YM cells in blueberry samples collected in the dumping area of packing lines [25]. The different observations between the two studies might be caused by multiple factors, such as the cultivar and climate differences, sample locations, and harvesting methods used. As stated previously, the Washington samples were collected from ‘Draper’ and ‘Liberty’ northern highbush blueberry plants, while the Georgia samples were collected from southern highbush (*V. corymbosum* unknown cvs.) and ‘Rabbiteye’ (*V. virgatum* unknown cvs.) blueberry plants. In addition to the cultivar and species variation, the blueberry samples from Georgia were collected at fresh blueberry packing facilities [25], while the ones from Washington were sampled in the field. Microorganisms can be introduced into blueberries through contaminated harvesting tools, containers, harvesters, gondolas, forklifts, or during the packing process [26]. Furthermore, the mean YM counts for the Washington samples presented in Table 2 were the mean populations of both the machine- and hand-harvested samples in the field. The Georgia samples were, nevertheless, all collected by machine harvesters—no handpicked fruit were included. This may be another contributing factor to the observed differences. Other than these factors, the hot and humid weather condition in the southeast and the possible waiting time between harvest and packing during the busy harvesting season could also make the microbial load of harvest blueberries relatively higher [27,28,29].

### 4.3. The Effect of Harvesting Time

The 9 am samples in the present study had significantly higher (*p* < 0.05) mean YM and TC counts than the 3 pm samples (Table 2). This could be explained by the temperature drops overnight and the water condensation on the surface of the fruit in the early morning hours of the day. These conditions may favor the survival and persistence of some microorganisms. Harvested fruit in berry lugs usually stay at the edge of the field for a period of time after harvest before being transported to refrigerated facilities. During this time, uncovered fruit may be exposed to solar radiation, which could be lethal to some microorganisms on the surface of the fruit [30]. These factors could explain why the YM and TC counts for the samples collected in the afternoon were relatively lower than those for the samples collected in the morning.

### 4.4. The Effect of Harvesting Methods

#### 4.4.1. Machine Harvesting

The samples of blueberries harvested using the conventional machine harvester in the current study had mean TA, YM, and TC counts of 2.30, 3.87, and 0.31 log CFU/g, respectively, and the counts for the samples of blueberries harvested using the modified machine harvester prototype were not significantly different (*p* > 0.05) from these values, except for the mean TC counts (Table 2). The samples of machine-harvested blueberries collected from the fields in other states in the United States seem to have relatively higher microbial counts according to the results of several previous studies. Popa et al. [31] reported mean TA, yeast, mold, and TC counts of 4.03, 4.32, 4.57, and 1.12 log CFU/g, respectively, for machine-harvested blueberries from fields in Michigan. The TA, yeast, and mold counts for the blueberries collected from lowbush blueberry (*V. angustifolium*) fields in Maine were reported to be within the range of 3.22 to 3.51, 2.27 to 2.72, and 2.46 to 3.35 log CFU/g, respectively [32]. The sampled Michigan blueberries were from ‘Bluecrop’ northern highbush blueberry, while the Maine blueberries were harvested from unnamed, ‘wild’ lowbush blueberry stands. These crops were grown under different environmental conditions and under different management compared to the fruit sampled in the present study. The production and postharvest handling practices likely varied as well, especially for the lowbush blueberry. The differences in the microbial counts for the fresh berries observed in the previous studies could be attributed to these factors.

Bruising is one of the biggest challenges associated with mechanically harvested blueberries. Studies have shown that the modified harvester prototype with soft catching surfaces usually maintains better firmness of harvested fruit compared to standard harvesters with hard surfaces, which ultimately enhances the shelf life [5,14]. It is encouraging to see that the TA counts for the samples harvested using the two different kinds of machines were not significantly different (*p* > 0.05; Table 2). However, the samples harvested using the modified harvester prototype did have significantly higher (*p* < 0.05) TC counts and a relatively more frequent presence of fecal coliforms. A higher level of coliforms can be an indication of unsanitary conditions, potentially related to contamination by polluted water, uncleaned surfaces, and employees with poor personal hygiene. Thus, preventative measures should be encouraged when the modified machine harvester is used to harvest fresh market fruit.

#### 4.4.2. Hand vs. Machine Harvesting

Regardless of the type of harvester, the microbial loads of the blueberries harvested using the machine harvesters were significantly higher (*p* < 0.05) than those of the blueberries harvested by hand (Table 2, Table 3 and Table 4). The most probable explanation is that machine harvesters may cause more internal bruises and external injuries to blueberries during harvesting, which accelerate the penetration and invasion of microorganisms into the fruit. A previous study observed higher incidences of plant diseases in machine-harvested blueberries during postharvest storage [33,34]. Blueberries can also be contaminated by microorganisms on the harvester surfaces if it is not appropriately cleaned or sanitized. The machine harvesters used in this study were cleaned and sanitized once a day in the late afternoon with water emitted from a high-pressure sprayer (Lisa Wasko DeVetter, personal communication). In comparison, the hand pickers were required to wash their hands whenever they started or returned to harvesting activities. The blueberry contact surfaces on the machine harvesters were thus not frequently cleaned nor sanitized, and this is a probable source of microbial contamination for the harvested blueberries.

Although the hand-harvested blueberries had relatively lower microbial loads, harvesting blueberries by hand may also project the risk of introducing human pathogens via the direct contact of the berries with contaminated hands. For example, *Staphylococcus aureus* is a typical type of pathogen that is usually carried by food handlers [35]. In the current study, fecal coliforms were found in some collected samples. Harvesting using gloved hands served as a control in this study. The other method of hand harvesting was done by following good agricultural, as well as appropriate hand washing, practices; therefore, the fruit harvested using this method had a similar microbial quality as the control. On machine harvesters, graders may also come into contact with fruit as they move along the inspection belt before falling into a lug. Graders and anyone who comes into direct contact with fruit pose the same risks as hand harvesters and should likewise follow good agricultural and handwashing practices. These results reemphasized the importance of having handwashing facilities in blueberry fields during the harvest seasons.

## 5. Conclusions

This study found that fruit harvested using the modified machine harvester prototype had similar (*p* > 0.05) TA and YM counts to fruit harvested using the conventional harvester, although the TC counts were higher for the fruit harvested using the modified harvester. However, the berry samples harvested using the two types of machine harvesters had significantly higher (*p* < 0.05) microbial loads than the handpicked samples, which emphasizes the importance of the routine cleaning and sanitation of machine harvesters. This research will benefit fresh berry and, perhaps, other fresh fruit producers.

## Figures and Tables

**Table 1:** Results of the type III tests for fixed effects by the statistical model of blueberry harvesting (α = 0.05).

	Effect	DF	Type III SS	Mean Square	F Value	Pr > F
TA	Cultivar	1	0.79	0.79	1.28	0.2589
	Sampling time	2	1.29	0.64	1.04	0.3558
	**Harvest method**	3	33.93	11.31	18.21	**<0.0001**
	Cultivar*time	2	0.86	0.43	0.69	0.5007
	Cultivar*method	3	0.57	0.19	0.31	0.8216
	Time*method	12	7.30	0.61	0.98	0.4679
YM	Cultivar	1	0.02	0.02	0.16	0.6914
	**Sampling time**	2	2.34	1.17	7.81	**0.0005**
	Harvest method	3	0.37	0.12	0.83	0.4775
	Cultivar*time	2	0.58	0.29	1.94	0.1457
	Cultivar*method	3	0.55	0.18	1.22	0.3012
	Time*method	12	1.65	0.14	0.92	0.5301
TC	Cultivar	1	0.68	0.68	1.06	0.3038
	Sampling time	2	3.47	1.74	2.70	0.0690
	**Harvest method**	3	8.63	2.88	4.47	**0.0043**
	Cultivar*time	2	0.70	0.35	0.55	0.5800
	Cultivar*method	3	2.90	0.97	1.50	0.2137
	Time*method	12	11.00	0.92	1.42	0.1541

TA: total aerobes; YM: total yeasts and molds; TC: total coliforms. DF: Degree of freedom. Pr > F: *p* value, which reflects the significance of the effect. A value smaller than 0.05 is a significant effect; *: Interaction between the two variables.

**Table 2:** Overall mean microbial load on the sampled fresh blueberries of various cultivars harvested using various methods at different sampling time points.

	Total Aerobes	Total Yeast and Molds	Total Coliforms
	log CFU/g		
*Cultivar*			
Draper (*n* = 192)	2.03 ± 1.02 ^A^	3.87 ± 0.47 ^A^	0.40 ± 0.90 ^A^
Liberty (*n* = 144)	2.10 ± 0.55 ^A^	3.85 ± 0.26 ^A^	0.36 ± 0.73 ^A^
*Sampling time*			
9 am (*n* = 120)	2.10 ± 0.83 ^A^	3.96 ± 0.36 ^A^	0.46 ± 0.85 ^A^
12 pm (*n* = 120)	2.12 ± 0.85 ^A^	3.86 ± 0.43 ^B^	0.41 ± 0.82 ^AB^
3 pm (*n* = 96)	1.95 ± 0.87 ^A^	3.73 ± 0.35 ^B^	0.25 ± 0.78 ^B^
*Harvesting method*			
Modified OTR prototype machine harvester (*n* = 88)	2.49 ± 0.48 ^A^	3.90 ± 0.39 ^A^	0.68 ± 0.93 ^A^
OTR machine harvester (*n* = 72)	2.30 ± 0.52 ^A^	3.87 ± 0.31 ^A^	0.31 ± 0.75 ^B^
Ungloved sanitized hands (*n* = 88)	1.81 ± 1.00 ^B^	3.80 ± 0.37 ^A^	0.29 ± 0.81 ^B^
Hands with sterile gloves (*n* = 88)	1.70 ± 0.94 ^B^	3.86 ± 0.48 ^A^	0.24 ± 0.73 ^B^

Means of each experiment variable followed by different letters in the same column are significantly different (*p* < 0.05).

**Table 3:** Microbial load on sampled fresh blueberries harvested using different methods at individual sampling time points.

	Modified OTR Machine Harvester Prototype (*n* = 88)	Standard OTR Machine Harvester (*n* = 72)	Ungloved Sanitized Hands (*n* = 88)	Hands with Sterile Gloves (*n* = 88)
	log CFU/g			
*Total aerobes*				
9 am (*n* = 120)	2.65 ± 0.50 ^a^	2.37 ± 0.56 ^a^	1.66 ± 0.87 ^b^	1.80 ± 0.83 ^b^
12 pm (*n* = 120)	2.43 ± 0.34 ^a^	2.29 ± 0.30 ^a^	2.04 ± 1.08 ^ab^	1.74 ± 1.03 ^c^
3 pm (*n* = 96)	2.34 ± 0.57 ^a^	2.24 ± 0.64 ^a^	1.71 ± 0.98 ^b^	1.51 ± 0.92 ^b^
*Total yeasts and molds*				
9 am (*n* = 120)	4.03 ± 0.34 ^A^	3.98 ± 0.33 ^A^	3.88 ± 0.30 ^A^	3.98 ± 0.46 ^A^
12 pm (*n* = 120)	3.89 ± 0.47 ^AB^	3.90 ± 0.29 ^A^	3.79 ± 0.43 ^AB^	3.88 ± 0.47 ^AB^
3 pm (*n* = 96)	3.76 ± 0.32 ^B^	3.77 ± 0.27 ^A^	3.69 ± 0.34 ^B^	3.69 ± 0.45 ^B^
*Total coliforms*				
9 am (*n* = 120)	0.91 ± 1.04 ^a^	0.33 ± 0.66 ^b^	0.31 ± 0.74 ^b^	0.27 ± 0.78 ^b^
12 pm (*n* = 120)	0.76 ± 0.89 ^a^	0.44 ± 0.43 ^ab^	0.25 ± 0.96 ^b^	0.13 ± 0.69 ^b^
3 pm (*n* = 96)	0.26 ± 0.73 ^a^	0.41 ± 1.02 ^a^	0.14 ± 0.64 ^a^	0.14 ± 0.66 ^a^

According to the results in Table 1, the harvest method is a significant factor influencing only the total aerobic and coliform counts, and the sampling time is a significant factor influencing only the yeast and mold counts. Values of the variables followed by different uppercase letters in each column are significantly different (*p* < 0.05). Values followed by different lowercase letters in each row are significantly different (*p* < 0.05).

**Table 4:** Microbial loads on sampled fresh blueberries of different cultivars as affected by the harvesting methods.

	Modified OTR Prototype Machine Harvester (*n* = 88)	Standard OTR Machine Harvester (*n* = 72)	Ungloved Sanitized Hands (*n* = 88)	Hands with Sterile Gloves (*n* = 88)
		log CFU/g		
*Total aerobes*				
Draper (*n* = 192)	2.50 ± 0.53 ^a^	2.25 ± 0.60 ^a^	1.74 ± 1.28 ^b^	1.64 ± 1.16 ^b^
Liberty (*n* = 144)	2.47 ± 0.42 ^a^	2.41 ± 0.31 ^a^	1.89 ± 0.45 ^b^	1.76 ± 0.57 ^b^
*Total coliforms*				
Draper (*n* = 192)	0.54 ± 0.98 ^a^	0.33 ± 0.84 ^a^	0.35 ± 0.88 ^a^	0.39 ± 0.87 ^a^
Liberty (*n* = 144)	0.85 ± 0.84 ^a^	0.20 ± 0.55 ^b^	0.26 ± 0.72 ^b^	0.07 ± 0.43 ^b^

According to the results in Table 1, the harvesting method is a significant factor influencing only the total aerobic and coliform counts. Values in the same row followed by the same letters are significantly different (*p* < 0.05).

## Data Availability

Data will be provided upon reasonable request.
